# High consumption of commercial food products among children less than 24 months of age and product promotion in Kathmandu Valley, Nepal

**DOI:** 10.1111/mcn.12267

**Published:** 2016-04-15

**Authors:** Alissa M. Pries, Sandra L. Huffman, Indu Adhikary, Senendra Raj Upreti, Shrid Dhungel, Mary Champeny, Elizabeth Zehner

**Affiliations:** ^1^ Helen Keller International Asia Pacific Regional Office Phnom Penh Cambodia; ^2^ Consultant to Helen Keller International; ^3^ Helen Keller International Kathmandu Nepal; ^4^ Ministry of Health and Population Kathmandu Nepal; ^5^ Helen Keller International Washington D.C. United States of America

**Keywords:** Nepal, snack foods, infant and young child feeding, double burden

## Abstract

Commercially produced complementary foods can help improve nutritional status of young children if they are appropriately fortified and of optimal nutrient composition. However, other commercially produced snack food products may be nutritionally detrimental, potentially increasing consumption of foods high in salt or sugar and displacing consumption of other more nutritious options. Helen Keller International, in collaboration with the Nepal government, implemented a study to assess mothers' utilization of commercial food products for child feeding and exposure to commercial promotions for these products. A cross‐sectional survey was conducted among 309 mothers of children less than 24 months of age across 15 health facilities. Utilization of breastmilk substitutes was low, having been consumed by 6.2% of children 0–5 months of age and 7.5% of children 6–23 months of age. Approximately one‐fourth (24.6%) of children 6–23 months age had consumed a commercially produced complementary food in the prior day. Twenty‐eight percent of mothers reported observing a promotion for breastmilk substitutes, and 20.1% reported promotions for commercially produced complementary foods. Consumption of commercially produced snack food products was high at 74.1% of children 6–23 months. Promotions for these same commercially produced snack food products were highly prevalent in Kathmandu Valley, reported by 85.4% of mothers. In order to improve diets during the complementary feeding period, development of national standards for complementary food products is recommended. Nutritious snack options should be promoted for the complementary feeding period; consumption of commercially produced snack food products high in sugar and salt and low in nutrients should be discouraged.

**Key messages**

- As consumption of commercially produced complementary foods is common among children 6–23 months in Kathmandu Valley; establishment of quality control and standards for nutritive value is crucial.
- There is a need for nutrition interventions to address overconsumption of commercially produced snack food products high in sugar and encourage healthier, low‐cost, convenient snack food options.
- Introduction of foods to infants before 6 months is prevalent in Kathmandu. Behaviour change communication strategies to promote exclusive breastfeeding among caregivers should be strengthened.
- Promotion of commercially produced snack food products is widespread in Kathmandu Valley, while promotions for breastmilk substitutes and commercially produced complementary foods are less commonly reported.

## Introduction

Exclusive breastfeeding for the first 6 months of life with continued breastfeeding up to 2 years of age or beyond is the optimal course of feeding for infants and young children (WHO and UNICEF 2003, 2015). A child's nutritional needs increase around the age of 6 months; it therefore becomes necessary to introduce complementary foods in a timely, safe and adequate manner, while continuing to breastfeed. This period commonly corresponds to growth faltering in young children and is an important focus area for preventing future childhood malnutrition (Shrimpton 2001). In Nepal, growth impairment occurs primarily during the first 2 years of life, a period when adequate nutrient intake is needed to avoid lifelong effects of malnutrition (HKI Nepal 2010). Despite improvements in the nutritional status of children in Nepal over the last 15 years, 41% of Nepalese children less than 5 years of age are stunted; 11% are wasted, and 39% are underweight (MOHP 2012).

Previous studies have found that complementary feeding practices in Nepal should be improved. Early introduction of complementary foods, which has the potential to negatively affect a child's nutritional status by displacing breastmilk in the diet and increasing illness, is common in Nepal; three percent of breastfed children aged 2–3 months receive some kind of solid or semi‐solid food, a figure which rises to 23% by 4–5 months of age (MOHP 2012). With regard to infant and young child feeding (IYCF) minimum standards, which account for dietary diversity, feeding frequency and consumption of breastmilk, milk or other milk products, only 37% of children in urban households and 24% of Nepalese children overall are fed in accordance with recommended practices (MOHP 2012).

The 2006 Nepal Demographic and Health Survey (NDHS) illustrated that 21% of infants 6–8 months of age, 35% of those 9–11 months of age and 55% of those 12–23 months of age ate sugary snack foods in the previous day (MOHP 2007), and a survey of young children in 16 districts across Nepal by the International Food Policy Research Institute reported consumption of savory snacks, such as chips or *chanachur* (crispy lentil‐based snack), and sweet snacks to be 52.0% at 9–11 months and 63.5% at 21–23 months of age (Cunningham 2013). These consumption patterns are cause for concern; often, these food products are energy dense and nutrient poor and are inappropriate for infant and young child feeding. Commercially produced complementary foods can help improve nutritional status of young children if they are appropriately fortified and of optimal nutrient composition (Lutter *et al*. 2008; Phu *et al*. 2012). However, other commercially produced snack food products may be detrimental to young child feeding by potentially increasing consumption of foods high in salt or sugar and displacing consumption of other more nutritious options. While previous studies have gathered information on consumption of ‘sugary snack’ foods, the prevalence of consumption of a greater range of specific snack products often eaten by young children, such as biscuits, candy, soft drinks and savory chips/crisps, needs to be better documented in order to inform infant and young child‐feeding policies and interventions. Therefore, the objective of this study was to assess consumption of commercial food and beverage products among children less than 24 months of age in Kathmandu Valley and build the understanding around mothers' exposure to commercial promotions for these products.

## Materials and methods

### Research design and study population

This cross‐sectional survey among mothers of children less than 24 months of age residing in Kathmandu Valley used a multistage sampling procedure to obtain a representative sample. Data were collected through structured interviews with mothers who were utilizing child health services at a facility. The survey was facility based because another objective of this study was to assess mothers' exposure to commercial promotion of infant and young child food products within the Kathmandu Valley health system; according to the 2011 NDHS, 90.0% of urban Nepal children 12–23 months of age were fully immunized, indicating high utilization of health facilities for child health services among caregivers of children less than 24 months of age (MOHP 2012). Mothers were asked to recall feeding practices for the youngest child and exposure to commercial promotions since the birth of their youngest child. Data were gathered from a period of December 2013 to February 2014.

Because prior studies have indicated commercial products to be more widely available in urban areas (Huffman *et al*. 2014), the study populations included in this survey were limited to mothers currently living in and utilizing health facilities within Kathmandu Valley, defined as the geographical area within the limits of Kathmandu, Lalitpur and Bhaktapur districts. Mothers living outside of Kathmandu Valley, but utilizing child health services in the Valley, were excluded. For ethical reasons, mothers with children too ill to be interviewed were also excluded. Additionally, mothers with any of the following characteristics were excluded because these conditions held the potential to delay breastfeeding initiation: mothers of children born with congenital diseases or who were in the neonatal intensive care unit, mothers who experienced severe delivery complications during the birth of their youngest child and mothers whose youngest child is a twin or from a multiple birth.

### Sample size

The sample size for this study was calculated to detect prevalence of 10% of children 6–23 months consuming commercially produced food on the preceding day and 10% prevalence of exposure to promotions, with a measurement error of ±5%. Using a standard of error of 0.0255 and assuming a design effect of 2 to account for the cluster design, a sample size of 280 mothers was determined. Because of the cluster‐sampling design utilized (described later), the final sample size was slightly higher than 280, with 309 mothers interviewed. A total of 435 mothers utilizing child health services at a facility were approached for interview. Fifty‐four (12.4%) of these mothers refused participation, and 72 (16.6%) mothers were excluded; 56 (12.9%) mothers lived outside of Kathmandu Valley, and 12 (2.8%) infants had been in the neonatal intensive care unit after delivery. Six (1.4%) mothers reported severe complications during delivery and one (0.2%) mother whose child was too ill for interview. The majority of refusals by mothers were due to the mothers not having time and needing to leave the health facility after their child received services. The final sample of successfully completed interviews was 309 mothers.

### Sampling procedure and data collection

Approximately 28% of urban Nepali mothers with facility‐based deliveries deliver at private facilities (MOHP 2012). In order to reflect this, 30% of the total samples of mothers were interviewed at private facilities. Rates of refusal were higher at private facilities as compared with public (17.2% vs. 10.3%, respectively), while rates of exclusion were higher at public facilities as compared to private (19.6% vs. 9.7%, respectively).

Lists of all public and private health facilities offering child health services were obtained from the Health Management Information System database from the District Public Health Offices for the three districts in Kathmandu Valley. This included national hospitals, referral hospitals and health centres; health posts were excluded. In addition, the same data source provided utilization rates for these facilities, which included total number of child health visits between July 2012 and July 2013, including outpatient department and immunization. Health facilities were sampled by allocating clusters using probability proportional to size based on utilization rates. Because of logistics requirements and the need to complete data collection within 8–10 weeks, facilities with less than 50 child health visits per month were excluded from the sampling frame. This excluded 26 out of 92 child health facilities; however, the 66 included in the sampling frame represented 98.3% of all child health visits in Kathmandu Valley health facilities that occurred between July 2012 and July 2013.

**Figure 1:**
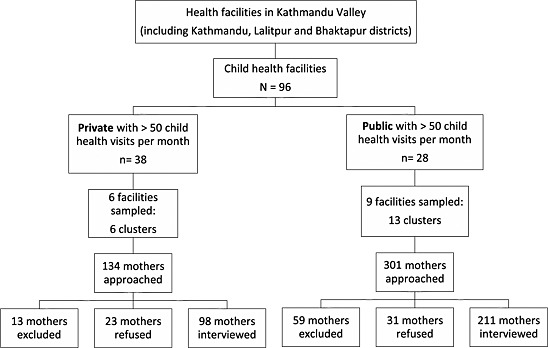
Sampling profile for mothers and facilities.

Clusters of 16 mothers each were assigned across facilities in the sampling frame; the total of 16 mothers per cluster was chosen to allow for even distribution of child ages across four age categories (0–5.9, 6–11.9, 12–17.9 and 18–23.9 months). Thirteen clusters were sampled in each sampling frame of public facilities, and six clusters were sampled for private facilities, allowing for 30% of the total sample to be sourced from private facilities. Figure 1 details the sampling of facilities and mothers for each study population across public and private health facilities.

Staff at sampled facilities were alerted of data collection approximately 1 week prior to survey. At each site, survey supervisors screened every woman with a child within the child health clinic area, including both immunization clinics and the paediatric outpatient department. Women were first screened to assess (1) if they were the mother of the youngest child brought to the clinic, (2) if this child was under 24 months of age and (3) if they lived in Kathmandu Valley. Survey supervisors also assessed the age of the child to verify if an interview was still needed for the specific age category.

Approval for this study was obtained from the Nepal Health Research Council prior to data collection. 1Approval was obtained on 2 December 2013 (Registration No. 156/2013). Informed consent was obtained from all participants prior to the conducting of any interview.

### Questionnaire design

The questionnaire gathered data regarding mothers' and children's characteristics, including mother's age, marital status, caste, educational attainment, household assets and drinking water source, and details regarding antenatal care and delivery of the youngest child and the child's date of birth, gender and birth order. Data on breastfeeding practices, current feeding practices and dietary intake of the youngest child were also collected. Data to assess infant and young child feeding practices were gathered in accordance with the World Health Organization guidelines on IYCF practices (WHO 2008). Standardized questionnaires were used to obtain information on which foods and liquids were consumed by the youngest child on the day and night prior to the day of interview. Additionally, data were gathered on the weekly frequency of consumption, reasons for feeding and expenditure for home‐prepared complementary foods and commercially produced snack food products. Mothers were also asked to report on observation of promotional practices for commercially produced food products since the delivery of their youngest child.

Data were collected using mobile devices in order to allow for immediate data entry, reduction in data errors and prompt analyses. The questionnaires were designed in Microsoft Word and then entered in Formhub, an open‐source online platform that allowed data to be collected via tablets, using the Android application Open Data Kit Collect, and data were then submitted online to a web‐based database (Formhub.org 2013). The questionnaires were translated from English into Nepali, back translated into English to ensure accuracy, and uploaded into Formhub in Nepali. Interviews were conducted in Nepali using the Samsung Galaxy tab 2.07 model tablet. Submitted questionnaires were reviewed weekly to ensure data quality.

### Statistical analyses

Data were cleaned and analysed using spss version 21 (IBM, Armonk, NY, USA). Proportions and mean ± standard deviation were used to describe the sample. Differences in age categories and associations were assessed through bivariate comparison, using two‐sided Pearson's chi‐squared test for proportions.

Commercial promotions were defined as any type of marketing technique intended to increase sales, including media or print advertising, provision of free samples, sales/discounts, coupons or any other activity to encourage or induce the purchase of a product (IBFAN 2007). Exposure to such commercial promotion was measured by asking mothers if they had heard, seen or read any promotions and if they had received any free samples, coupons or discounts for products since the birth of their youngest child. These questions were adapted from the Interagency Group on Breastfeeding Monitoring's protocol for Estimating the Prevalence of Violations of The Code (IGBM 2007). They were comprehensively pre‐tested to ensure that the definitions of ‘promotion’ and the products referenced were clearly understood by respondents. Additionally, respondents were asked to recall the brands of products promoted, and these were cross‐referenced to ensure that the brands reported were in fact products within these food product categories. Utilization of commercial food products was measured by mother's reported feeding of a product to her youngest child in the day prior to interview.

## Results

### Demographics and socio‐economic characteristics

Demographic and socio‐economic characteristics for mothers and children are shown in Table 1. The majority of all mothers were currently married at the time of interview, and almost two‐thirds (59.9%, *n* = 185) of mothers reported their child under 24 months of age to be their only child. Among those that were currently married, 13.6% (*n* = 42) of mothers reported that their husband currently worked outside of Kathmandu. Eighty‐nine percent (*n* = 275) of mothers had attended any level of formal education; 21.7% (*n* = 67) of mothers reported attending university or higher graduate studies. Only 9.7% (*n* = 30) of mothers reported currently working outside the home, and almost all mothers (95.5%, *n* = 295) reported themselves to be the main caregiver of their youngest child less than 24 months of age.

**Table 1:** Demographic and socio‐economic characteristics of mothers and children (*n* = 309)

Mother	
Age (years) (mean ± SD)	26.0 ± 4.7
Parity (number) (mean ± SD)	1.5 ± 0.7
Marital status (%)	
Married	99.7
Divorced, widowed or separated	—
Single	0.3
Level of education (%)	
None	7.1
Non‐formal education	3.9
Primary	12.0
Secondary	28.5
Upper secondary	26.9
Tertiary education	21.7
Caste (%)	
Dalit	3.2
Disadvantaged janajati	24.6
Disadvantaged non‐dalit terai caste	2.9
Religious minority	0.3
Advantaged janajati	23.0
Upper caste	46.0
Works outside the home (%)	9.7
Main caregiver of child (%)	95.5
Received antenatal care (%)	99.0
Assisted delivery (%)	95.5
Child	
Age (mean ± SD)	11.6 ± 6.8 (months)
Sex (female) (%)	48.9
C‐section delivery (%)	28.2
Household	
Safe source of drinking water (%)	97.7
Household members per sleeping room (mean ± SD)	2.7 ± 1.1
Assets, ownership (%)	
Bicycle	18.1
Car	5.5
Motorbike	47.2
Refrigerator	43.0
Television	90.6

SD, standard deviation.

The mean age of children less than 24 months of age was 11.6 months, as would be anticipated given the effort made to sample children across an equal distribution of ages 0–23 months. Almost all mothers (99.0%, *n* = 306) had received some antenatal care during pregnancy with their youngest child. Almost one‐third (28.2%, *n* = 87) of the children referenced in this survey were delivered by caesarean section, and most mothers (95.5%, *n* = 295) delivered their youngest child with the assistance of a health professional.

Comparison of these characteristics to the 2011 NDHS indicates that the mothers included in this study are of a slightly higher socio‐economic status than the general population of women in urban Nepal. Seventy‐seven percent of mothers in this study had achieved an educational level of secondary school or higher, as compared with 63.7% of urban Nepal women, and only 7.1% of mother included in the survey had never attended school, as compared with 22.0% of women living in urban Nepal (MOHP 2012). Household assets were also greater among mothers included in this survey. Nearly half of mothers (47.2%) reported that their household owned a motorbike, 43.0% a refrigerator and 90.6% a television, as compared with 27.8%, 29.3% and 76.2% of urban Nepal women, respectively (MOHP 2012).

Mothers attending private facilities reported higher rates of caesarean deliveries as compared with mothers attending public facilities; 37.8% of those seeking child health care in private facilities reported their youngest child was delivered via C‐section, as compared with 23.7% of those seeking child health care in public facilities (*P* = 0.014).

### Infant and young child feeding practices

In addition to gathering information on exposure to promotional practices for commercial food products, mothers were asked about current infant and young child feeding practices, including breastfeeding and complementary feeding practices; results are shown in Tables 2 and 3. Almost all children had ever been breastfed, and the majority was currently breastfeeding at the time of interview. Exclusive breastfeeding was practiced by 39.5% (*n* = 32) of mothers of children less than 6 months of age, while 59.3% (*n* = 48) were predominantly breastfeeding their child less than 6 months of age. Over one‐third of children under 6 months (39.5%, *n* = 32) were given plain water in the previous day, and 24.7% (*n* = 20) were given a soft, semi‐soft or solid food, which increased with the age of the infant: 9.4% at 0–1 months, 23.3% at 2–3 months and 52.6% at 4–5 months (*P* = 0.002). Rice/cereals were given by all mothers who reported feeding a food to their child less than 6 months of age. Bottle feeding was practiced among just over one‐third of mothers of children less than 24 months of age (34.6%, *n* = 107), with a greater proportion of bottle feeding occurring among children over 6 months as compared with children less than 6 months of age (43.0% vs. 11.1%; *P*‐value < 0.001). Twenty‐two percent of all children drank plain water from a bottle; 7.4% drank milk, and 6.5% drank commercial water. Four and a half percent drank infant formula, and 1.6% drank chocolate/malt‐based drinks (mothers could have reported if their child drank more than one liquid from a bottle).

**Table 2:** Percentage of mothers with types of current breastfeeding and bottle feeding practices

	*n*	%
Ever breastfed	309	97.4
Currently breastfeeding		
0–5 months	81	98.8
6–11 months	78	97.4
12–17 months	77	96.0
18–23 months	73	88.6
Exclusively breastfeeding†, ‡	81	39.5
Predominantly breastfeeding†, §	81	59.3
Continued breastfeeding at 1 year¶	55	92.7
Continued breastfeeding at 2 years∥	43	79.1
Bottle feeding,		
0–5 months	81	11.1
6–11 months	78	43.6
12–17 months	77	39.0
18–23 months	73	46.6

† Among children 0–5 months.

‡ Defined as an infant that received only breastmilk in the 24‐h preceding interview, with the exception of ORS, drops or syrups (vitamins, minerals and medicines) allowed (WHO 2008).

§ Defined as an infant that received breastmilk in the last 24‐h preceding interview, and certain liquids, including water, water‐based drinks, fruit juice, ritual liquids, oral rehydration solution (ORS) and drops or syrups (vitamins, minerals, medicines). Any other foods or liquids (non‐human milk and food‐based fluids) are not allowed (WHO 2008).

¶ Among children 12–15 months.

∥ Among children 20–23 months.

**Table 3:** Percentage of children 6–23 months of age meeting minimal complementary feeding indicators (*n* = 228)

Minimum dietary diversity, %†	57.5
Minimum meal frequency, %‡	78.1
Minimum acceptable diet, %	51.3

† Calculated based on World Health Organization infant and young child feeding indicators; minimum dietary diversity was defined as consumption of at least four out of seven food categories (WHO 2008).

‡ Calculated based on World Health Organization infant and young child feeding indicators; minimum meal frequency was defined as at least two times for breastfed children 6–8 months, at least three times for children 9–23 months and at least four times for non‐breastfed children 6–23 months (WHO 2008).

Complementary feeding practices were assessed based on dietary intake information among children 6–23 months of age. Minimum dietary diversity, defined as a child consuming at least four of seven food categories in the previous day (WHO 2008), was met by 57.5% (*n* = 131) of children 6–23 months of age. Minimum meal frequency, defined as a child consuming food the minimum number of times or more in the previous day depending on their age and breastfeeding status (WHO 2008), was met by 78.1% (*n* = 178) of children 6–23 months of age. A minimum acceptable diet, defined as the combination of these two indicators (WHO 2008), was achieved by only half (51.3%, *n* = 117) of children 6–23 months of age. Children of mothers with higher socio‐economic characteristics were more likely to have achieved a minimum acceptable diet. Sixty‐nine percent of children of mothers who had attained a university/graduate level of education had been fed a minimum acceptable diet, as compared with 46.4% of children of mothers who had not attended university (*P* = 0.006), and 56.9% of children of mothers who were from privileged societal groups (including advantaged Janajati or other upper castes) had been fed a minimum acceptable diet as compared with 38.2% (*P* = 0.014) of children of mothers from non‐privileged societal groups (including Dalit, disadvantaged Janajati, disadvantaged non‐Dalit Terai caste and religious minorities).

The practice of adding a sweetener, such as sugar or honey, to liquids or foods fed to young children was common among mothers interviewed for this study. Almost half (46.1%, *n* = 105) of mothers of children 6–23 months reported adding a sweetener to a liquid their child drank, and 37.3% (*n* = 85) of mothers reported adding sugar or honey to their child's food. Over half of mothers (52.2%, *n* = 119) of children 6–23 months of age reported adding a sweetener to either a food or a liquid consumed by their child in the day prior to interview.

### Consumption of breastmilk substitutes among children less than 24 months of age

Mothers were asked to report on their youngest child's consumption of breastmilk substitutes in the day preceding the interview. Overall, consumption of breastmilk substitutes among children less than 24 months of age was low, reported among only 7.1% of all mothers (*n* = 22). Rates of breastmilk substitute consumption during the exclusive breastfeeding period were low, with only 6.2% (*n* = 5) of children below 6 months of age having been fed this in the last day. Nine percent of children aged 6–11 months of age (*n* = 7), and 6.7% of children ages 12–23 months (*n* = 10) had consumed a breastmilk substitute in the day prior to interview.

### Consumption of commercial complementary foods and home‐made complementary foods among children 6–23 months of age

**Figure 2:**
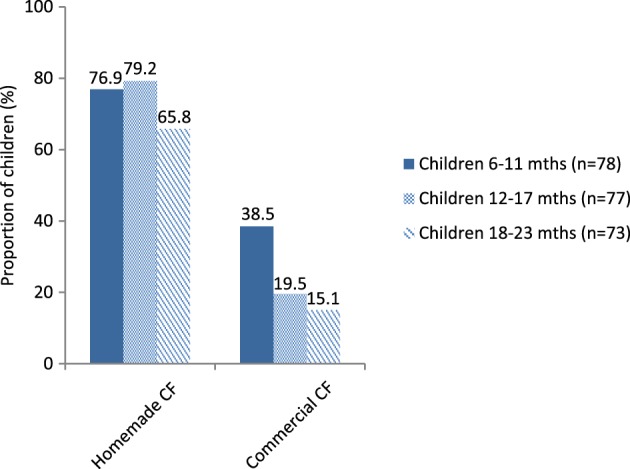
Percentage of children 6–23 months of age consuming home‐made/commercially produced complementary food, by age category.

Rates of consumption of commercially produced and home‐made complementary foods among children 6–23 months of age are shown in Fig. 2. Of children 6–23 months, 74.1% (*n* = 169) had consumed a home‐made complementary food. The majority of children 6–23 months of age (86.8%, *n* = 198) had consumed a home‐made complementary food in the week prior to interview, and 61.8% (*n* = 141) of mothers reported feeding a home‐made complementary food to their child every day in the past week. The most common home‐made complementary foods included *jaulo* (rice and lentils cooked together), which was consumed by 59.8% of those who ate home‐made complementary foods, *n* = 101, *lito* (roasted grain/lentil flour cooked in water or milk), consumed by 37.3% (*n* = 63) and mashed rice with lentils, consumed by 32.5% (*n* = 55). Nearly all of these home‐made complementary foods contained cereals (94.1%), 66.3% contained lentils and 43.8% contained a fat source (butter, oil or ghee). The majority (84.0%, *n* = 142) of mothers who fed their child a home‐made complementary food in the day prior to interview reported that they fed this food because it was ‘healthy’. The rates of feeding home‐made complementary foods were similar between working and non‐working mothers (76.5% and 76.9%, respectively). Consumption of home‐made complementary foods was similar across age groups among children 6–23 months of age (*P* = 0.111).

Consumption of commercially produced complementary foods was less common than home‐made complementary foods, with 24.6% (*n* = 56) of children 6–23 months age having consumed a commercially produced complementary food in the day prior to interview. Three‐fourths (76.8%, *n* = 43) of the children consuming a commercially produced complementary food had consumed a commercial infant cereal, either the imported Cerelac brand or locally manufactured commercial *sarbottham pitho (*flour often made of soybean, wheat and maize and sometimes fortified). One‐third of working mothers (32.4%, *n* = 11) reported feeding their youngest child a commercially produced complementary food, as compared with one‐fourth (24.5%, *n* = 45) of non‐working mothers; this difference was not statistically significant (*P* = 0.393). A significantly greater proportion (38.5%, *n* = 30) of children 6–11 months of age were consuming commercially produced complementary foods, as compared with children over 12 months of age [17.3% (*n* = 26); *P* = 0.001].

### Consumption of commercially produced snack food products and beverages for general consumption among children 6–23 months of age

**Figure 3:**
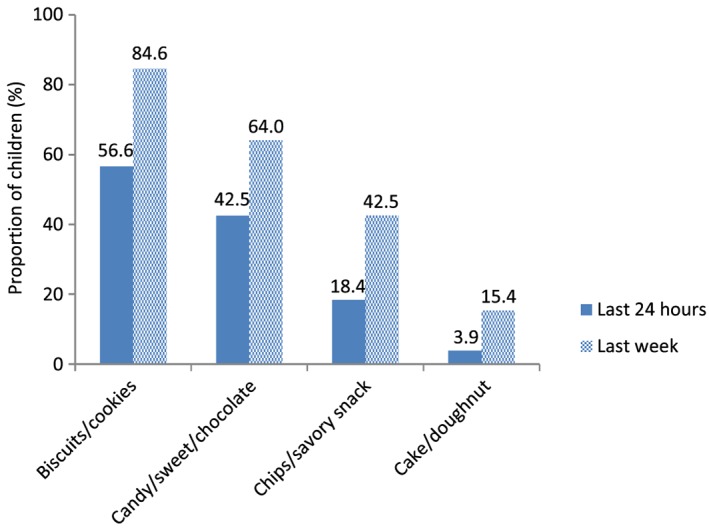
Percentage of children 6–23 months of age consuming snack foods 6–23 months within the last 24 h and last week (*n* = 228).

Mothers were asked to report on their youngest child's consumption of commercially produced snack food products for general consumption that may be commonly fed to young children, but not specifically marketed to young children; this included cookies/biscuits, savory snacks, cakes/doughnuts and candy/sweets/chocolate. Consumption of these foods in the day and week prior to interview among children 6–23 months of age is shown in Fig. 3.

Overall, 74.1% (*n* = 169) of children 6–23 months of age consumed a commercially produced snack food product on the day prior to interview, and almost all (91.2%, *n* = 208) had consumed one in the week prior to interview. The most highly consumed commercially produced snack food products were sugary snack foods. Over half (56.6%, *n* = 129) of children 6–23 months consumed a biscuit/cookie, and nearly half (42.5%, *n* = 97) had consumed a candy/chocolate/sweet in the previous day. Approximately one‐third (37.7%) of children had consumed one of these four snack food product types (biscuits/cookies, savory snacks, cakes/doughnuts and candy/sweets/chocolate) on the day prior to interview; while 26.8% had consumed two types, 8.3% had consumed three product types, and 1.3% had consumed all four product types. In the week prior to interview, biscuits and cookies were consumed daily by almost one‐third of children 6–23 months of age (30.2%, *n* = 58), and candy or chocolate was eaten daily by 16.9% (*n* = 24) of children. For both these food products, consumption rates increased with age. Almost half of children 6–11 months of age (46.2%, *n* = 36) consumed a commercial biscuit or cookie in the previous day, as compared with 57.1% (*n* = 44) of children 12–17 months and 67.1% (*n* = 49) of children 18–23 months of age; this difference in consumption by age groups was significant (*P* = 0.034). For consumption of candy or chocolate, 21.8% (*n* = 17) of children 6–11 months had eaten this in the previous day, as compared with 44.2% (*n* = 34) of children 12–17 months and 63.0% (*n* = 46) of children 18–23 months; this difference in consumption of candy and chocolate by age categories was significant (*P* < 0.001).

Data were also gathered on mothers' reasons for feeding various commercially produced snack food products. Across all snack products, the majority of mothers reported feeding the product to their youngest child because ‘the child likes it’ (data not shown). The second most commonly reported reason was because the mother reported it to be a convenient food to feed, being reported by 40.6% (*n* = 78) of mothers feeding biscuits/cookies and 33.8% (*n* = 48) of mothers feeding candy/sweets/chocolate. Seventeen percent (*n* = 39) of all mothers interviewed reported avoiding feeding commercially produced snack food products to their children because they believed children become sick or develop a cough after eating them. However, 69.7% (*n* = 27) of mothers who reported avoiding feeding commercially produced snack food products to their children had fed such a product to their child in the day prior to interview. Mothers who reported purchasing these products in the last week reported spending 8.3 NPR ($US0.09) 2
http://www.xe.com/currencyconverter/convert/?Amount=1&From=NPR&To=USD, Friday, 18 July 2014, $US1 = 96.4500 NPR per day on cookies/biscuits, 4.7 NPR ($US0.05) per day on candy/chocolate, 6.8 NPR ($US0.07) per day on chips/savory snacks and 5.3 NPR ($US0.05) on cakes/doughnuts. Eighty‐six percent of children of mothers from non‐privileged societal groups had consumed a snack food product, as compared with 72.9% of children of mothers from privileged societal groups (*P* = 0.036). Eighty percent of children of mothers who had not attended university had consumed a snack food product on the previous day, as compared with 66.7% of children of mothers who had attended university; this was borderline significant (*P* = 0.081).

Mothers were also asked to report their youngest child's consumption of sugar‐sweetened commercially produced beverages, including soft drinks, juice drinks and chocolate/malt‐based drinks. Only one child had consumed a soft drink, and six children had consumed juice drinks on the day prior to interview. Thirteen percent (*n* = 40) of all children had consumed a chocolate/malt‐based drink during the previous day, with consumption increasing significantly with age; 3.7% of children 0–5 months and 6.4% of children 6–11 months had consumed a chocolate/malt‐based drink, while consumption occurred among 15.6% of children 12–17 months and 27.4% of children 18–23 months (*P* < 0.001).

### Exposure to promotion for commercial food and beverage products: breastmilk substitutes, complementary foods, and snack foods

In addition to gathering information on current infant and young child feeding practices, mothers were asked about exposure to promotional practices for commercial food products since the birth of their youngest child; results are shown in Table 4.

**Table 4:** Percentage of mothers exposed to promotions for commercial foods and beverages commonly consumed by children < 24 months of age (*n* = 309)

	Mothers with children <24 months (*n* = 309)
Observed promotion for breastmilk substitute	27.5
Received discount/coupon for breastmilk substitute	1.0
Received free sample of a breastmilk substitute	0.0
Observed promotion for commercial complementary food	20.1
Received discount/coupon for commercial complementary food	1.0
Received free sample of a commercial complementary food	1.0
Observed promotion for commercially produced snack food product	85.4

**Figure 4:**
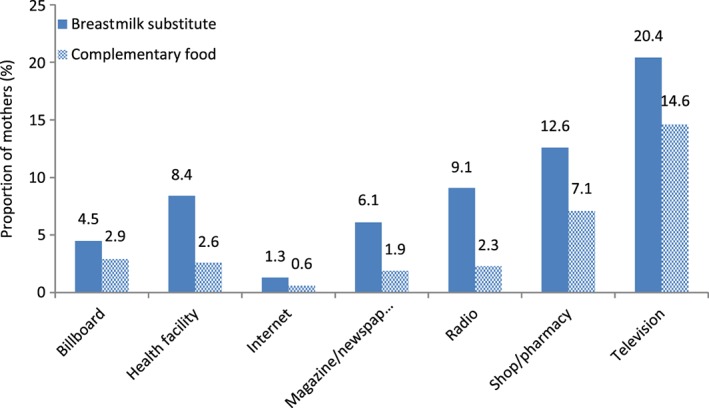
Percentage of mothers who reported hearing, seeing or viewing promotions for breastmilk substitutes and commercially produced complementary foods by location of promotion, (*n* = 309).

Over one‐quarter (27.5%, *n* = 85) of mothers reported observing a commercial promotion for a breastmilk substitute. Twenty percent (*n* = 62) of mothers reported observing a promotion for a commercially produced complementary food;14.6% (*n* = 45) of mothers reported observing a promotion for Cerelac infant cereal, and 4.9% (*n* = 15) reported observation of a promotion of other infant cereals, most commonly commercially produced *sarbottam pitho*. Six mothers (1.9%) also reported promotions for malt‐based drinks as falling in the category of ‘commercial drinks or foods specifically for infants and young children.’ While a prior study that analysed labels of some malt‐based drinks available in Kathmandu Valley showed that they do not specify feeding for children less than 24 months of age (Pereira *et al*. 2014), these mothers considered these products as specifically for their young children. The locations of reported promotions for breastmilk substitutes and commercially produced complementary foods are shown in Fig. 4. Television was the most commonly reported source of promotions for both categories, cited among 20.4% (*n* = 63) of mothers for breastmilk substitute promotions and 14.6% (*n* = 45) of mothers for commercially produced complementary food product promotions.

In addition to observation of commercial advertisements for IYC food products, mothers were also asked about exposure to other promotional practices sometimes employed by manufacturers, including receipt of free samples, discounts or coupons. Reported exposure to these other promotional practices was very low. No mothers reported receiving any free samples of breastmilk substitutes, and three mothers (1.0%) reported receiving a free sample of a complementary food. Only 1.0% (*n* = 3) of mothers reported receiving a discount or coupon for a breastmilk substitute or a commercially produced complementary food product.

**Figure 5:**
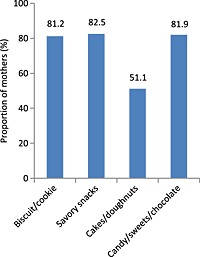
Percentage of mothers who reported commercial snack food promotions by type of food, (*n* = 309).

Reported promotions for commercially produced snack food products for general consumption were highly prevalent; 85.4% percent (*n* = 264) of mothers reported observing such a promotion. The levels of reported promotions for specific types of commercially produced snack food products are shown in Fig. 5.

## Discussion

Commercially produced snack food products, often of limited nutritional value, are being regularly consumed by children 6–23 months of age in Kathmandu Valley, Nepal, as well as being pervasively marketed according to mothers' reports. These findings carry implications for child health and nutritional outcomes, including overweight/obesity and undernutrition among young children and increased risk for non‐communicable diseases. Findings also indicate that consumption of breastmilk substitutes was low among children less than 24 months of age and continued breastfeeding was common among mothers interviewed.

Early introduction of complementary foods is prevalent among infants in Kathmandu Valley; 53% of children 4–5 months of age included in this survey received soft/semi‐soft/solid food in the day prior to interview, a higher rate than the 23% of 4–5‐month‐olds noted in the 2011 NDHS (MOHP 2012). In our study, most foods consumed by these young infants were similar to foods eaten by older infants and young children, including mixtures of grains, beans/lentils, a fat source and sugar or honey. Seventeen percent (*n* = 14) of children below 6 months had consumed a home‐made complementary food in the day prior to interview, while 6.2% (*n* = 5) had consumed a commercially produced complementary food. A recent study found that most (86%) labels of commercial complementary food products sold in Kathmandu Valley appropriately recommend introduction from the age of 6 months (Pereira *et al*. 2014); however, efforts are recommended to ensure all labels of commercially produced complementary foods state introduction at 6 months of age.

In addition to displacing breastmilk during the vital exclusive breastfeeding period and increasing the risk for poor nutritional outcomes, a recent review of the evidence indicated the potential association of very early introduction (before 4 months of age) of complementary foods and overweight/obesity (Pearce *et al*. 2013). Continued and strengthened messaging regarding the timely introduction of complementary foods may encourage exclusive breastfeeding until 6 months of age. One study in Nepal showed sufficient knowledge among mothers to be the most influential factor in regards to optimal feeding practices, and whether or not mothers received advice on IYCF practices during immunization visits was associated with appropriate feeding (Chapagain 2013). Secondary analysis of the most recent Nepal DHS revealed that mothers who attended four or more antenatal care visits were more likely to feed their children in accordance with recommended frequency (Khanal *et al*. 2013). Additionally, a review of strategies aimed to improve complementary feeding found that educational interventions improved feeding practices and had a positive impact on child growth within populations that were food secure (Imdad 2011). Ensuring delivery of IYCF information and counselling during routine child health visits could support timely introduction of complementary foods.

Results from the indicators of quality of diet for children 6–23 months in this study were of concern. Only half of children 6–23 months of age in this study achieved a minimum acceptable diet. Improving the quality of home‐made complementary foods to include more animal products and to be more convenient could be an important intervention to improve child nutritional status. Additionally, given the increased risk of infection (Black *et al*. 1989), efforts should be made to reduce the high rates of bottle feeding among infants and young children in Kathmandu Valley.

Commercially produced complementary foods are consumed by a significant proportion of young children in Kathmandu Valley; 24.6% of all children 6–23 months of age had consumed such a food in the previous day. Currently, Nepal does not have a national standard for processed fortified complementary foods, and these foods do not require compulsory certification. Given the prevalent consumption of these products among children 6–23 months of age, establishment of a strong quality control mechanism with mandatory certification is crucial to ensure that commercially produced complementary foods meet food and nutrition safety standards. Codex standards for formulated complementary foods could be used to inform national standards in Nepal, as well as be used in the interim as national legislation is being drafted (Codex Alimentarius 1991). Advocacy around standards and monitoring in regards to production of fortified complementary foods may also play an important role.

Among children included in this study, consumption of commercially produced snack food products was highly prevalent. Three‐fourths of children 6–23 months of age had consumed a commercially produced snack food product in the day prior to interview, mainly sweetened snacks including biscuits, candy or chocolate. This rate is higher than that reported for urban areas (61%) in Nepal in the 2006 NDHS (Huffman *et al*. 2014). In addition to high consumption of sugary snacks, over half of mothers of children 6–23 months of age also reported adding a sweetener, such as sugar or honey, to liquids or foods consumed by their child. World Health Organization guidelines recommend limiting sugar intake among children to less than 10% of a child's total energy intake (WHO 2015); quantities of free sugar intake were not measured in this survey and merit further investigation. Such high rates of sugar and sugary snack food consumption are of grave concern because of the relationship with dental caries (Moynihan 2005), and the increased risk for overweight/obesity and later development of chronic disease (Morenga *et al*. 2013; Malik *et al*. 2010; Malik *et al*. 2013). A study of 5–6‐year‐olds in Kathmandu Valley found that 69% had dental caries (Subedi *et al*. 2011), and reduction of sugar intake is an important means to reduce such high caries prevalence (Moynihan & Petersen 2004).

Nutritional interventions targeting reduced consumption of sugary snack foods are essential in Kathmandu Valley. While iron‐fortification of the refined flour commonly used to produce cookies/biscuits is mandatory within Nepal, the fortification level is not designed to meet the specific needs of young children, and these products are also often high in sugar content. Reduction in free sugar intake is vital to reduce chronic diseases such as diabetes, which are of increasing concern in Nepal (Bhandari *et al*. 2014). Additionally, the prevalence of overweight and obesity is increasing in children younger than 5 years globally and is a contributor to diabetes and other chronic non‐communicable diseases in adulthood (Black *et al.* 2013). According to 2011 NDHS, 1.4% of all children under 5 years in Nepal and 1.9% of children under 5 years in the Central Hill region (including Kathmandu Valley) are overweight or obese. While rates are low among children, adult rates are high; 13.5% of women in Nepal are overweight/obese, and 22.2% of women in Central Hill region are overweight/obese (MOHP 2012). A number of studies have shown the association between malnutrition in early life and obesity risk later in life (Black *et al.* 2013; Huh *et al.* 2011). Additionally, taste and food preferences can be established during the complementary feeding period (Beauchamp and Mennella 2009), with evidence indicating that early and repeated consumption of sugary/sweet foods is associated with increased intake of sugary/sweet foods later in life (Ventura and Mennella 2011; Avena *et al*. 2008). Consumption of unhealthy foods during the complementary feeding period could serve as a pathway to development of obesity and non‐communicable diseases during adulthood (Adair 2012).

In addition to being commonly fed to young children in Kathmandu Valley, promotions for these commercially produced snack food products are more highly prevalent than promotions for either breastmilk substitutes or commercially produced complementary foods. In this study, the association between exposure to promotion of snack food products and consumption among children 6–23 months was investigated; no statistically significant association was found. The sample size for this study was calculated to assess prevalence of consumption, and we hypothesize that it is not large enough to establish an association. Additionally, because the prevalence of mothers' exposure to promotions for commercially produced snack food products and children's consumption of these products is both high, this results in a particularly small sample for comparison; only 14.6% of mothers did not observe a promotion, and only 25.9% of children 6–23 months did not consume a commercially produced snack food product on the prior day. However, numerous prior studies have shown the impact of promotion on children's food preferences and consumption (Boyland *et al*. 2011; Andreyeva *et al*. 2011; Jones and Kervin 2010). Because promotion of energy‐rich and nutrient‐poor food products can foster rapid weight gain and increase the risk of chronic disease and obesity in children (Goris *et al.* 2009), especially those showing poor linear growth (Lobstein *et al*. 2015), addressing the high rates of promotion and consumption of sugary snack foods should be a national priority. Governments have used various methods to address exposure of caregivers and young children to advertising of unhealthy food products and to limit the impact of this advertising on consumption behaviours, (Harris *et al*. 2009). Regulations that directly restrict advertising of these products have shown some impact on reducing advertisements and product utilization. In 2008, South Korea passed legislation that restricts televised food advertising to children, and the country has seen some reduction in advertisements (Kim *et al*. 2013). Researchers assessing a ban on fast‐food advertising in Canada found that these restrictions contributed significantly to reductions in fast‐food expenditures (Dhar and Baylis 2011). Regulation of marketing is one mechanism the Nepal government could enact to counter high rates of promotion and consumption of commercially produced snack food products among children.

There is also a need for nutrition interventions in Nepal to improve the quality of diet among children 6–23 months of age in Kathmandu Valley in order to prevent increases in childhood overweight and obesity. This includes encouraging the replacement of unhealthy snacks with more nutritious, convenient foods, and ensuring that nutrition messaging provides advice to mothers on what are and are not nutritious snack options. Additionally, these snacks must be nutritious, convenient *and* affordable. According to the 2011 Nepal Living Standards Survey, average daily food expenditure among urban Nepal families is 349.26 NPR ($US3.44) (Central Bureau of Statistics 2011). Reported costing data from this study indicates that Kathmandu Valley mothers spend minimal amounts on commercial snack food products for their children in comparison with their total daily food expenditure. The associations also found between certain characteristics of lower socio‐economic status and children's consumption of these snack food products may be partly because these mothers are pressured to choose an inexpensive snack option for their children.

Consumption of unhealthy snack food products among young children is increasing globally. Demographic and Health Surveys in various countries, including Nepal, have captured data on consumption of ‘sugary snacks’ among young children, but a variety of snack food products are often consumed by young children. Comprehensively measuring snack product consumption, including sugary snacks, savory snacks and sweetened beverages, through national surveys like the DHS would allow consumption patterns to be monitored, nationally and globally and would also facilitate evaluation of programmes and policies aimed at reducing overweight/obesity. It is important to attempt to reduce the amount of sugar added to all foods and beverages fed to young children; overconsumption of unhealthy foods early in life can displace consumption of other important micronutrients and contribute to not only childhood overweight/obesity but also undernutrition (Anderson *et al*. 2008; Vartanian *et al*. 2007). Efforts to ensure adequate nutrition for infants and young children would serve to combat malnutrition during childhood, with the potential to positively impact adult overweight/obesity and associated non‐communicable diseases.

## Source of funding

This research was funded by the Bill & Melinda Gates Foundation.

## Conflicts of interest

The authors have no conflicts of interest to declare.

## Contributions

AP analyzed the data and prepared the manuscript. SH and EZ conceptualized and designed the study, with input from SU. MC oversaw questionnaire development and technology for data collection. IA and SD managed data collection. All authors reviewed and provided input on the final article.
